# Combating autophagy is a strategy to increase cytotoxic effects of novel ALK inhibitor entrectinib in neuroblastoma cells

**DOI:** 10.18632/oncotarget.6778

**Published:** 2015-12-28

**Authors:** Sanja Aveic, Marcella Pantile, Anke Seydel, Maria Rosaria Esposito, Carlo Zanon, Gary Li, Gian Paolo Tonini

**Affiliations:** ^1^ Neuroblastoma Laboratory, Pediatric Research Institute, Città della Speranza, Padua, Italy; ^2^ Ignyta Inc., San Diego, California, USA

**Keywords:** neuroblastoma, autophagy, ALK inhibitors, drug combination, entrectinib

## Abstract

Neuroblastoma (NB) is a threatening childhood malignancy. Its prognosis is affected by several morphological, and biological characteristics, including the constitutive expression of ALK tyrosine kinase. In this study we examined the therapeutic potential of a novel ALK inhibitor, entrectinib, in obliterating NB tumor cells.

Entrectinib showed the growth-inhibitory effects on NB cells with a 50% inhibitory concentration range of 0.03–5 μM. In the ALK-dependent cells, entrectinib mediated G1-arrest, which was associated with modified expression of multiple cell-cycle regulators. Down-regulation of *Ki-67*, and attenuated phosphorylation of ERK1/2, and STAT3, correlated with observed antiproliferative capacity of entrectinib. Initial cytostatic activity of entrectinib was followed by concentration-dependent apoptotic cell death, and Caspase-3 activation. However, we delineated a reduced sensitivity of *ALK* mutated NB cells to entrectinib, and demonstrated strong activation of autophagy in SH-SY5Y^F1174L^ NB cell line. Abrogation of autophagy by chloroquine increased significantly the toxicity of entrectinib, as confirmed by enhanced death rate, and PARP protein cleavage in SH-SY5Y^F1174L^ cells.

In aggregate, our data show that entrectinib inhibits proliferation, and induces G1-arrest, and apoptosis in NB cells. We propose entrectinib for further consideration in treatment of NB, and recommend pharmacological inhibition of autophagy to be explored for a combined therapeutic approach in NB patients that might develop resistance to entrectinib.

## INTRODUCTION

Neuroblastoma (NB) is one of the most common cancers in children under 1 year of age, and accounts for 15% of all pediatric cancer mortality [1]. While some NBs respond strongly to treatment or even regress spontaneously without cure, there are still a considerable number of NB cases in which current therapy is ineffective [2]. Some clinical, and molecular biological factors correlate with outcome of NB patients, and are defined as prognostic markers of this disease [3]. *MYCN* gene amplification has been observed in about 20% of all NB cases, and represents one of the strongest markers associated with the aggressiveness of the disease [4]. Increased expression of other genes, such as *TrkA*, and *caspase-1/-3/-8*, were described to correlate with favorable prognosis, whereas increased expression of *TrkB*, and *Survivin*, were associates with unfavorable outcome [5–7]. More recently, the *ALK* (anaplastic lymphoma kinase) gene has been found mutated in about 8% of sporadic NB, and has been associated with rapid disease progression [6–8].

*ALK* gene encodes for a cell surface neural receptor tyrosine kinase (RTK) which is dominantly expressed in developing embryonic, and neonatal brain [9]. The *ALK* mutations are shown to give the proliferative advantages to the cells in which they occur [10], and the constitutive activation of *ALK* gene has been found to give a particular negative impact over prognosis of NB [11]. The *ALK*^F1174L^, and *ALK*^R1275Q^ are the most frequent mutations found in sporadic NBs, leading to a single amino acid substitution in the tyrosine kinase domain (TKD) of ALK receptor [12]. Similarly to other RTK, these mutations cause a constitutive activation of ALK receptor, and downstream ALK-dependent regulatory pathways, giving a crucial impact over NB oncogenesis. Taken together, these findings introduced the possibility of targeting ALK protein in NB patients. During recent years, several compounds have been proposed for the treatment of patients with deregulated ALK protein, showing a diverse efficiency, and specificity [12, 13]. Nevertheless, the obstacle related to the use of ALK inhibitors in NB therapy remains their low efficiency in impairment of *ALK*-mutated NBs' growth. This problem is usually correlated with the acquired resistance of tumor cells, which prevents the successful treatment of NB patients [14, 15]. Hence, it becomes necessary to screen for a more successful therapeutic approaches, to overcome current therapy restrictions.

Here, we tested a novel ALK inhibitor entrectinib (RXDX-101) (Ignyta, San Diego) for its capacity to abrogate growth of NB cell lines with different status of *ALK* gene (wild type, mutated or amplified) *in vitro*. We showed that entrectinib was able to impair *ALK*-amplified (*ALK^amp^*) cells' growth, and proliferation, whereas its activity was less effective in NB cell lines bearing *ALK* mutation (*ALK*^F1174L^, and *ALK*^R1275Q^), or wild type (*ALK^wt^*) gene. We investigated for a mechanism possibly involved in low efficiency of entrectinib in *ALK*-mutated cells, and observed that autophagy induction after entrectinib addition was responsible for drug resistance. Chemical inhibition of autophagy increased significantly the efficiency of entrectinib in *ALK*-mutated cells, suggesting a combined therapy as a promising approach in patients with *ALK*^F1174L^ or *ALK*^R1275Q^ positive tumors.

## RESULTS

### Entrectinib blocks proliferation of NB cells

**Figure 1:**
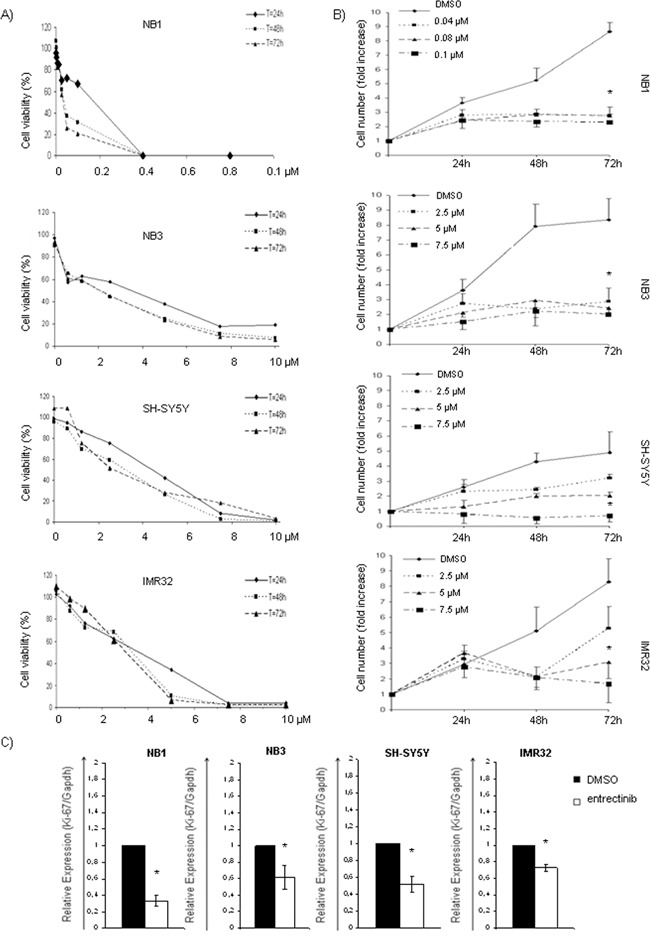
Inhibition of cell viability, and growth after entrectinib treatment **A.** NB cells were treated with increasing concentrations of entrectinib for 24h, 48h and 72h, and IC_50_ has been calculated for each cell line (NB1, NB3, SH-SY5Y and IMR32) for three time points. Concentrations used (μM) are indicated on X-axis. **B.** Trypan blue exclusion assay has been done to count the cell number in presence of three different concentration of entrectinib (2.5; 5; 7.5 μM). Data represent fold change of cell number calculated with respect to the number of cells present in the moment of administration of entrectinib, and expressed as mean ± SEM of 3 independent experiments. Time points are indicated on X-axis. **C.** Cell proliferation was measured by the means of *Ki-67* mRNA expression 24h after addition of entrectinib (NB1 = 0.08 μM; NB3, SH-SY5Y, IMR32 = 2 μM). Results were presented as a relative expression calculated with respect to DMSO control (RQ = 1). Significant down-regulation of *Ki-67* mRNA levels was evidenced for all cell lines. Single experiments were done in triplicates, and results presented for 3 separated treatments as mean ± SEM. Results were considered significant for *p ≤ 0.05.

To evaluate the concentration range activity of entrectinib, we treated NB1^amp^, NB3^R1275Q^, SHSY5Y^F1174L^, and IMR32^wt^ cells with increasing concentrations of entrectinib. The cell viability was checked after 24h, 48h, and 72h, by MTT assay. The inhibitory concentration of 50% (IC_50_) was calculated for each time point (Supplementary Table S1). Dose dependent cell viability reduction was observed in all cell lines. However, the cell viability was highly depressed in NB1^amp^ cells, which was extremely sensitive to entrectinib treatment (48h IC_50_ = 0.035 ± 0.009 μM). Sensitivity of other cell lines (NB3^R1275Q^, SH-SY5Y^F1174L^, IMR32^wt^) was also detected although it was lower with respect to NB1 cells (48h IC_50;_ NB3 = 2.24 ± 0.89 μM; SH-SY5Y = 3.32 ± 0.90 μM, IMR32 = 3.29 ± 0.35 μM; Figure 1A). Subsequently, we looked whether entrectinib induces cell morphology changes. We observed dramatic cell shape changes since the cells became smaller in size. Moreover, NB cells greatly reduced the capacity to become confluent (Supplementary Figure S1). A capacity of entrectinib to reduce NB cells' proliferation was validated in following. Concomitantly, after 24h of treatment, the cells showed a significant proliferative block that was directly proportional to the concentration of entrectinib used, and was maintained until 72h of treatment (Figure 1B; Supplementary Table S2).

Decreased proliferative capacity of entrectinib treated NB cells was confirmed by evaluating *Ki-67* nuclear antigen, an important marker of cell proliferation [16], by Real Time quantitative PCR (qRT-PCR). We confirmed a significant decreasing of *Ki-67* mRNA 24h after treatment with entrectinib, particularly in NB1, NB3, and SH-SY5Y cell lines, and in a lesser extent in IMR32 cells (DMSO control: RQ = 1; RQ of treatments: NB1 = 0.34 ± 0.06, *p* = 0.0005; NB3 = 0.62 ± 0.14, *p* = 0.05; SH-SY5Y = 0.52 ± 0.08, *p* = 0.006; IMR32 = 0.73 ± 0.05, *p* = 0.004; *n* = 3; Figure 1C). These results have been confirmed by immunocytochemistry, demonstrating the increased fraction of Ki-67 negative entrectinib-treated cells particularly in NB1 cell line, and less marked expressional changes of Ki-67 protein in IMR32 cells (Supplementary Figure S2).

### Entrectinib induces block in G1-phase of cell-cycle

**Figure 2:**
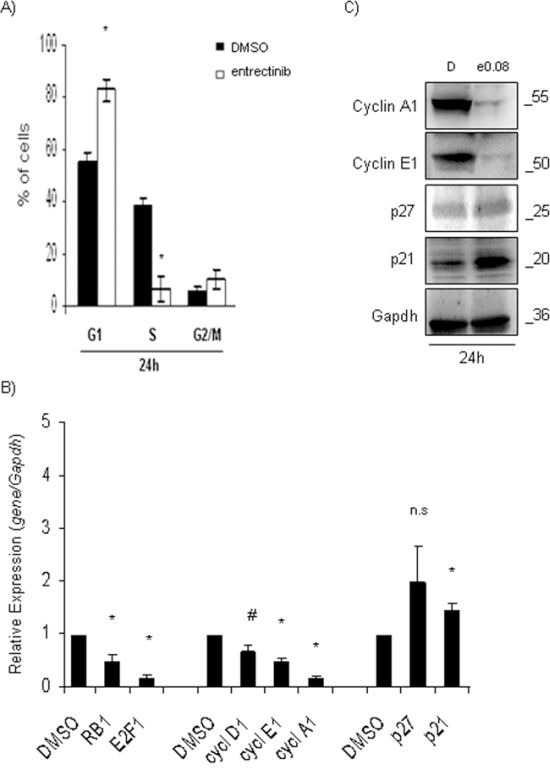
Entrectinib modifies cell-cycle profile **A.** Cell-cycle profile was evaluated by flow cytometry. Percentage of cells in each phase of cell-cycle is presented. An important accumulation of the NB1 cells in G1-phase was confirmed 24h post-treatment. **B.** qRT-PCR was performed for the evaluation of mRNA levels of the main cell-cycle regulators *p21, p27, Cyclin A1, D1, E1, pRB1,* and *E2F1*, after treatment with entrectinib (0.08 μM). Data were normalized for *Gapdh* internal control, and calculated with respect to the relative expression determined for DMSO control samples. Results were considered significant for p ≤ 0.05, and indicated with an asterisk (*). #*p* = 0.06. **C.** Expression of cell cycle regulatory proteins (p21, p27, Cyclin A1, and Cyclin E1) was validated by Western Blot 24h after treatment with entrectinib. Gapdh was used as a control of proper protein loading. Numbers indicate concentration of entrectinib (e; μM) used. D = DMSO.

Observed proliferative reduction of NB cells was caused in part by cell-cycle inhibition, as confirmed by propidium iodide staining analysis. The cell-cycle distribution in NB1 cells, treated with a single concentration of entrectinib (0.08 μM), demonstrated a significant accumulation in G1-phase after 24h, with respect to control (G1, 24h: DMSO = 55.4 ± 3.0 %; entrectinib = 83.0 ± 4.3 %; *n* = 3, *p* = 0.006; Figure 2A). Additionally, a decrease of S-phase was confirmed (S, 24h: DMSO = 38.6 ± 2.8 %; entrectinib = 6.5 ± 4.4 %; *n* = 3, *p* = 0.004; Figure 2A). For the remaining 3 cell lines, a tendency of G1-arrest was observed even though a statistical significance was not reached for the concentration of entrectinib used (2.5 μM; Supplementary Figure S3A, S3B and S3C). Moreover, we examined the association between entrectinib-induced G1-arrest, and alteration of cell-cycle regulatory genes. We analyzed the expression contents of *p21, p27, Cyclin A1, Cyclin D1, Cyclin E1, RB1,* and *E2F1* genes, which are well-known cell-cycle regulators. Expression levels of *RB1, E2F1, Cyclin D1, Cyclin E1,* and *Cyclin A1,* were significantly reduced in entrectinib treated NB1 cells, whereas the contents of *p21,* and *p27* were markedly increased (Figure 2B, and Supplementary Table S3) when compared to control samples (DMSO: RQ = 1), mirroring the changes in cell-cycle distribution observed previously. The similar changes in genes' expression were found for the remaining NB cell lines (Supplementary Figure S3A', S3B' and S3C'). A block of NB1 cells in G1-phase has been confirmed by Western blot analysis as well, showing a particular accumulation of p21 protein 24h post-treatment with entrectinib (Figure 2C). Oppositely, levels of Cyclin A1, and E1 were down-regulated.

### Addition of entrectinib decreases the clonogenic capacity of NB cells

**Figure 3:**
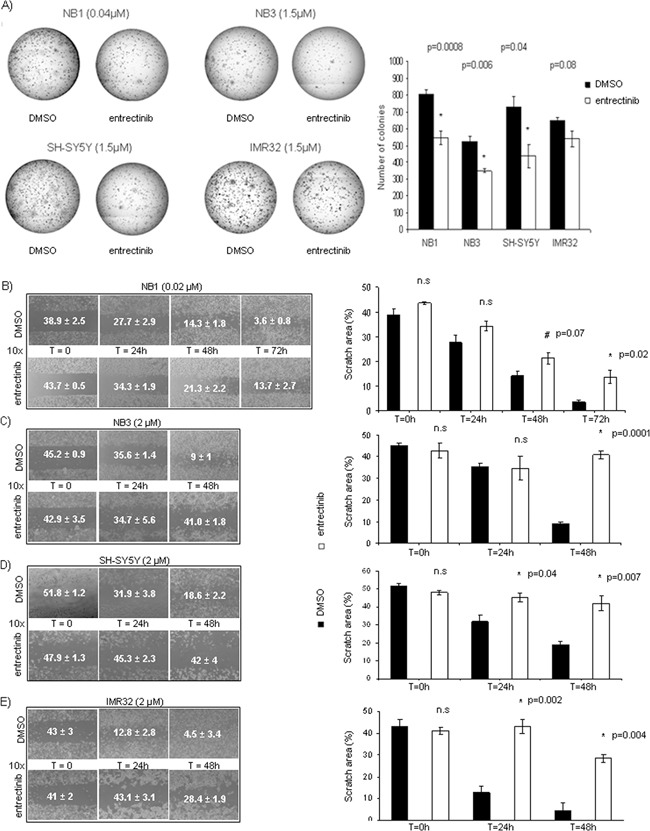
Entrectinib impairs NB cell clonogenic competence, and motility **A.** Representative images of clonogenic assays were shown for each cell line (left side). Cells were analyzed for the foci formation during the course of 14 days in the presence of either entrectinib or DMSO control vehicle. The figure shows the capacity of entrectinib to deteriorate the colony-forming cell. The number of colonies was counted by ImageJ software after coloration with MTT, and results shown as mean ± SEM (at right) of three independent experiments. Results were considered significant for *p < 0.05. Numbers within the brackets indicate the μM of entrectinib used in the experiment. **B-E.** Time-lapse microscopy reveals an inhibition of the motility of neuroblastoma cells upon entrectinib treatment (left side). For the wound healing assay, NB cells were treated with vehicle (DMSO control) and B) 0.02 μM (NB1) or C) 2 μM (NB3, SH-SY5Y, and IMR32) entrectinib, and monitored for 48-72h., The results are presented for each image (right side) as mean ± SEM of three separate experiments, and *p-value considered significant when ≤ 0.05. n.s.: not-significant; # - close to significance.

We seeded NB cells in methylcellulose in addition of entrectinib to investigate the possibility of entrectinib to impair NB cells to form colonies, and let them to proliferate for 14 days (Figure 3A, left panel). The entrectinib gave rise to significantly fewer, and smaller colonies with respect to control cells (colony number NB1: DMSO = 803.8 ± 29.0; entrectinib = 548.4 ± 40.0; *n* = 3; *p* = 0.001; NB3: DMSO = 522.7 ± 35.0; entrectinib = 348.7 ± 10.3; *p* = 0.006; *n* = 3; SH-SY5Y: DMSO = 727.0 ± 65.5; entrectinib = 437 ± 70.9; *n* = 3; *p* = 0.04; IMR32: DMSO = 648.5 ± 19.3; entrectinib = 541.5 ± 47.0; *n* = 3; *p* = 0.08; Figure 3A, right panel) in all cell lines tested. Together, these results indicated that entrectinib had the capacity to inhibit NB cells to form the colonies.

### Entrectinib reduces motility of NB cells

The time-lapse microscopy of NB cells after treatment with entrectinib revealed an inhibition of the motility of NB1, NB3, SH-SY5Y, and IMR32 cells upon entrectinib treatment. In the scratch assay, DMSO control cells were able to migrate into the scratch area, whereas treatment with entrectinib prevented wound closure. The scratch area (expressed as percentage of total area; Figure 3B, 3C, 3D and 3E, left images) significantly decreased for the control cells, whereas it remained largely uncovered in the presence of entrectinib (Figure 3B, 3C, 3D and 3E, right graph bars). We also observed differences in the velocity of treated cells to repopulate the scratch zone compared to controls. In particular, a complete closure was reached after 48h for IMR32, 72h for SH-SY5Y, and NB3, and after 96h for NB1 (data not shown). Our results clearly demonstrated the strong anti-migratory effects of entrectinib on NB cells with different *ALK* gene status.

### Entrectinib triggers cell death in NB cell lines

**Figure 4:**
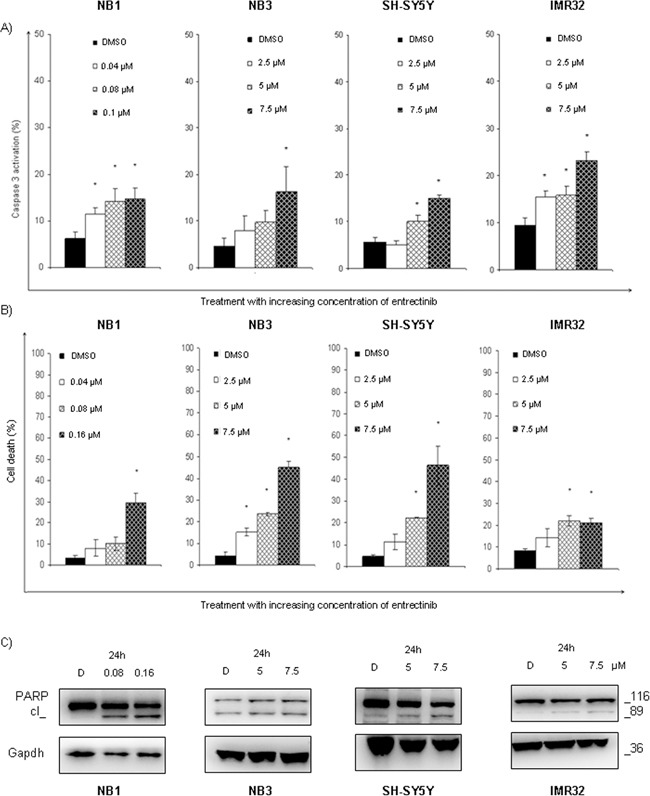
Entrectinib stimulates cell death in NB cell lines **A.** The capability of entrectinib to induce Caspase-3 dependent death of NB cells was assessed applying flow cytometry technique. An increase in Caspase-3 activation was detected in different extents for diverse cell types 24h after treatment with increasing concentration of entrectinib. NB1^amp^ was particularly sensitive to entrectinib, whereas SH-SY5Y^F1174L^ showed weaker sensitivity to the compound. **B.** Percentage of dead cells was measured by TUNEL assay, applying flow cytometry technique. The potential of entrectinib to impair cell viability was confirmed for all cell lines, for different concentration of entrectinib. The effects of entrectinib, and DMSO treated control samples are presented. Results were considered significant for *p ≤ 0.05. **C.** Activation of PARP protein, as a hallmark for death induction, was seen by Western blot. Cleavage of PARP protein was most evident for NB1 cell line. Gapdh was used as protein loading control. D = DMSO.

To assess the possible induction of apoptosis, we treated NB cells with increasing concentrations of entrectinib. A significant increase in Caspase-3 activation was found for NB1 cell line, and in comparable extent for NB3, SH-SY5Y, and IMR32 cells (Figure 4A; Supplementary Table S4; *n* = 3; p < 0.05). A dosage-dependent increase of dying cells was also observed by TUNEL assay (Figure 4B; Supplementary Table S5; *n* = 3; p < 0.05). At the protein level, cell death induction was confirmed by studying the expression of the early marker of apoptosis poly (ADP-ribose) polymerase (PARP), which was fragmented proteolytically by Caspase-3. An evident cleavage of PARP protein occurred due to entrectinib activity as shown in Figure 4C. Collectively, these results imply for entrectinib to be a potent inhibitor of NB cells growth, which is hampered by cell-cycle blocking, and inducted apoptosis. Other hallmarks of apoptosis, including mitochondrial membrane depolarization, and generation of reactive oxygen species, were not stimulated after use of IC_50_ dose of entrectinib (data not shown).

### Entrectinib blocks ALK-dependent signaling pathway

**Figure 5:**
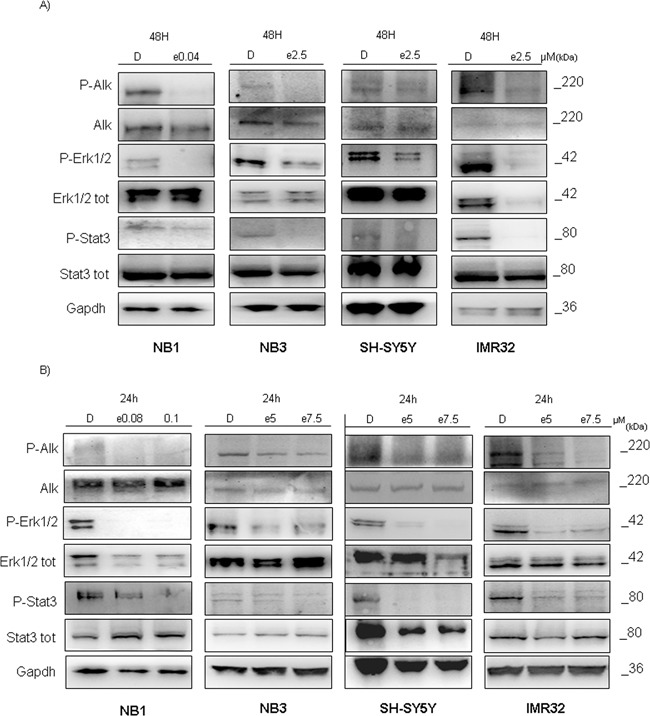
Entrectinib impacts ALK downstream protein pathway Eventual change of ALK downstream pathway after treatment with entrectinib was validated by Western blot. Two different analyses were done: **A.** using entrectinib (e) IC_50_ defined for 48h, and **B.** using increasing concentration of entrectinib for 24h. Decreased activity of two principal downstream target of ALK protein, ERK1/2, and STAT3 can be seen by their reduced phosphorylation in either of immunoblots presented. Gapdh was used as a control of proper protein loading. Numbers beside letter (e) indicate concentration of entrectinib (μM) used. D = DMSO.

The observed results prompted us to evaluate for the possible changes in ALK-downstream signaling pathway after addition of entrectinib. Abrogation of ALK function by adding entrectinib (IC_50_ or increasing concentrations), led to a decreased expression of pERK1/2, and pSTAT3 proteins, two principal regulators of cell proliferation, in concentration, and time dependent manner (Figure 5A and 5B). Similar effects were seen for all NB cell lines tested, supporting the proliferative advantages of the NB cells with deregulated ALK function.

### Autophagy induction in SH-SY5Y cell line explains the low entrectinib efficiency

**Figure 6:**
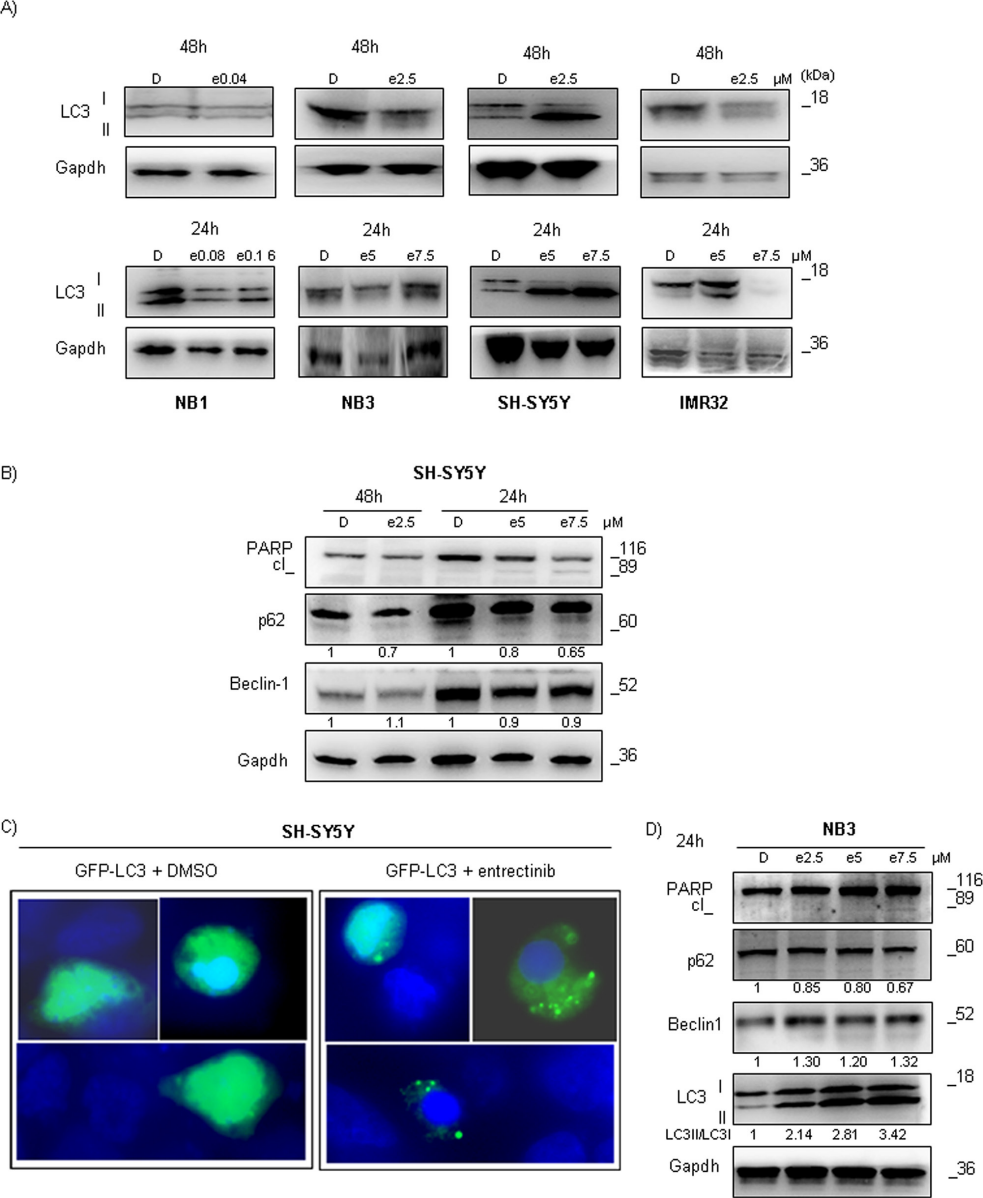
Entrectinib causes autophagy activation in ALK mutated NB cells **A.** Immunoblot analyses of LC3 after treatment with IC_50_ (upper lane) or increasing concentration (lower lane) of entrectinib are presented. A significant processing of LC3 was seen for SH-SY5Y cells with *ALK* ^F1174L^ mutation. Gapdh was used as a protein loading control. **B.** Apoptosis (PARP), and autophagy (p62, and Beclin-1) related proteins were examined in SH-SY5Y cell line for several concentrations of entrectinib (e), and for 2 time points, 24h and 48h. Gapdh was used as a control of proper protein loading. Numbers beside letter (e) indicate concentration of entrectinib (μM) used in the experiments. Numbers alone indicate relative expression of the protein calculated by densitometry. D = DMSO. **C.** SH-SY5Y cells were transiently transfected with GFP-LC3 plasmid, and treated with entrectinib (5 μM) or DMSO vehicle for 24h. Fluorescence microscopic analysis was done afterwards to evaluate a distribution of GFP-LC3 within the cells. Cell nuclei were counterstained with DAPI (blue). Images were taken on a confocal microscope (100X immersion objective), equipped with a digital camera. **D.** Apoptosis (PARP), and autophagy (LC3, p62, and Beclin-1) related proteins were examined in NB3 cell line for increasing concentration of entrectinib. Gapdh was used as a control of proper protein loading. Initial numbers indicate concentration of entrectinib (e) in μM. Numbers under the blots indicate relative expression of the protein calculated by densitometry. D = DMSO.

*ALK^F1174L^* is considered one of the most aggressive *ALK* mutations in NB, and shows high transforming potential [17]. In view of the poorer efficiency of entrectinib to induce cell death in ALK mutated NB cells, particularly SH-SY5Y^F1174L^, we speculated that some protective mechanism could be activated, contributing to the observed scenario. Autophagy represents the most common adaptive cellular response in cancer that can enhance the tumor cell survival during treatments [18]. One of the most specific indicators of autophagosome formation, and hence autophagy activation, is a microtubule associated protein 1 light chain 3 (LC3) [19, 20]. At first, we checked how the levels of LC3-I, and LC3-II changed after addition of entrectinib. The expression of LC3-II protein was significantly induced after treatment with entrectinib for 48h (Figure 6A, upper lane), and was more evident with increase of entrectinib concentration (24h; Figure 6A, lower lane). This event was particularly marked in SH-SY5Y cells, and was confirmed by calculating the LC3-II/LC3-I ratio, demonstrating a clear prevalence of LC3-II levels in treated samples with respect to DMSO controls (LC3-II/LC3-I ratio: DMSO = 1 Arbitrary Units; entrectinib 5 μM = 5.4 AU; entrectinib 7.5 μM = 5.6 AU; Figure 6A, lower lane). Moreover, our results revealed the degradation of autophagic marker p62/SQSTM1 (Figure 6B) [21]. Interestingly, Beclin-1 levels, as an apical regulator of autophagy, did not increase significantly after treatment with diverse entrectinib concentrations, implying that the mechanism of autophagy activation in SH-SY5Y was likely independent of Beclin-1, as reported in other studies as well [22].

To further confirm that the autophagy was induced in SH-SY5Y cells after addition of entrectinib, we performed a transient transfection with GFP-LC3 plasmid. Under the fluorescence microscope, GFP-LC3-transfected cells treated with DMSO showed a diffuse distribution of green fluorescence, whereas entrectinib treatment (5 μM) triggered vesicle formation, creating a punctuated form of GFP-LC3 protein (Figure 6C). The diverse patterns confirmed that LC3 was engaged in autophagosome formation in entrectinib-induced autophagy in SH-SY5Y^F1174L^ cell line.

### Autophagy induction after entrectinib treatment was less obvious in NB3 cell line

Our data demonstrate that the NB3^R1275Q^ cells are slightly more sensitive to entrectinib than SH-SY5Y^F1174L^. To examine how different *ALK* mutations may affect induction of autophagy in response to entrectinib, we checked the expression of principal autophagy regulators in NB3 cells 24h post-treatment. Even in this cell line we could observe autophagy activation, although it was less marked in comparison to SH-SY5Y cells (Figure 6D). Beside decrease in p62 protein levels, increase in LC3-II/LC3-I ratio (LC3-II/LC3-I ratio: DMSO = 1 AU; entrectinib 2.5 μM = 2.14 AU; entrectinib 5 μM = 2.81 AU; entrectinib 7.5 μM = 3.42 AU), and marginal cleavage of PARP protein, we confirmed a slight up-regulation of Beclin-1 protein for NB3 cell line (Figure 6D). These results indicated that autophagy induction followed a different pattern in diverse NB cell lines, but contributed directly to the sensitivity of NB cells to entrectinib.

### Inhibition of autophagy potentiates entrectinib-stimulated cell death

**Figure 7:**
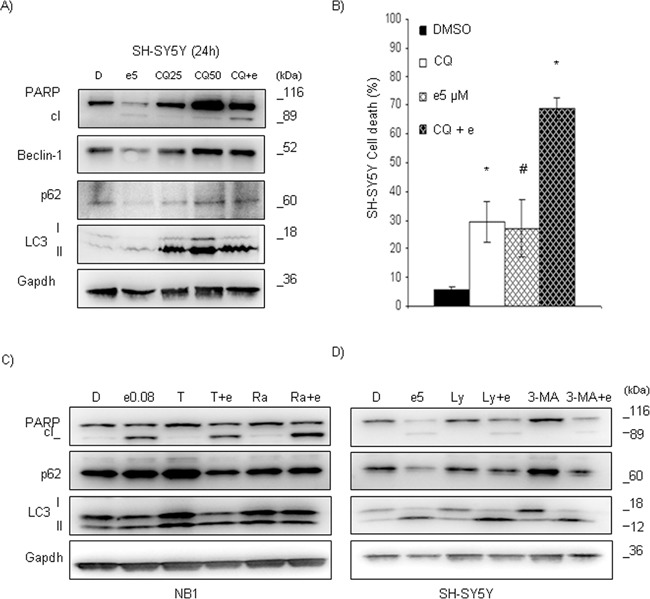
Entrectinib activity in SH-SY5Y cells increases after abrogation of autophagy **A.** Cells were pre-incubated with Chloroquine (CQ; 25 and 50 μM) for 1h, and entrectinib (e; 5 μM) was added in following for 24h. Whole cell lysates were used to evaluate the expression of PARP, p62, Beclin-1, and LC3 proteins for single or combined treatments. Gapdh was utilized as loading control. Numbers indicate concentration of compounds in μM used in the experiment. D = DMSO. **B.** Percentage of dead cells was measured by TUNEL assay, applying flow cytometry technique. The potential of entrectinib (e; 5 μM) to impair cell viability alone, or after pre-treatment with CQ (50 μM), was confirmed in SH-SY5Y cell line. The effects of treatments, and DMSO control samples are presented as mean ± SEM. Results were considered significant for *p ≤ 0.05. #*p* = 0.09. **C.** Tamoxifen (5 μM) and Rapamycin (10 μM) were used to stimulate autophagy in NB1 cells, whereas D) LY294002 (10 μM), and 3-MA (50 μM) were used as autophagy inhibitors in SH-SY5Y cells. Immunoblot analyses were performed to verify the levels of apoptosis- and autophagy-related proteins. Gapdh served as a loading control. Abbreviations: D, DMSO; e, entrectinib; T, Tamoxifen; Ra, Rapamycin; Ly, Ly294002; 3-MA, 3-Methyladenine. Numbers indicate concentration of entrectinib (μM) used for the experiments.

To examine autophagic flux in SH-SY5Y^F1174L^ cell line, we analyzed LC3-II in the presence of the lysosomal protease inhibitor chloroquine (CQ) [23]. We pre-treated SH-SY5Y cells with CQ (50 μM; for 1h), and subsequently added entrectinib (5 μM) to operate for 24h. Afterwards, proteins were extracted for Western blot analysis, while cell death was measured by flow cytometry (TUNEL assay). The dynamic autophagic process was confirmed after inhibition of lysosomal protease by CQ, through a notable accumulation of LC3-II, p62, and Beclin-1 proteins (Figure 7A). In addition, LC3-II levels increased further in CQ-entrectinib co-treated cells compared to control. These findings implied that the observed accumulation of autophagic vacuoles resulted from autophagy induction rather than from their decreased degradation by lysosomes [24]. More importantly, the effects of CQ, in CQ+entrectinib combined treatment, provoked an increase in cell death, as confirmed by both, Western blot (PARP activation, Figure 7A) analysis or TUNEL assay (DMSO = 5.8 ± 1.0; CQ = 29.4 ± 7.1; entrectinib = 27.0 ± 9.8; CQ+entrectinib = 68.8 ± 3.9; *n* = 3; p < 0.05; Figure 7B).

To explore whether the protective role of autophagy against entrectinib-induced cell death was cell specific, we chemically stimulated autophagy in NB1 cells by Tamoxifen (T), and Rapamycin (Ra), two well-known autophagic stimulators. After pre-treatment with T and Ra, cells were treated with entrectinib, and LC3-II, and p62 levels were examined after 24h. Even though both compounds showed a capacity to increase LC3 cleavage (LC3-I), and modification (LC3-II), the final effects were somewhat different (Figure 7C). In particular, Tam lead to an increase of p62 levels, which drastically diminished in combination with entrectinib, implying for an enforcement of autophagic flux (Figure 7C, lane 3 and 4). Treatment with Rapa did not change p62, and LC3 levels, but did increase a cleavage of PARP protein when used in combination with entrectinib, suggesting for enforced apoptosis activation. These results suggested that NB1 cells survival was highly dependent on ALK activity, which may be considered as the main pathway that gave them the proliferative benefits. This also indicates that entrectinib affects autophagic flux, most probably at LC3-II level by sequestration into autophagosome. The opposite experiment performed on SH-SY5Y cells, using other autophagy inhibitors which are known to act upstream of phagophore formation (*e.g.* PI3K inhibitor LY294002 (Ly), and 3-Methyladenine (3-MA)) supported these findings. Once again, entrectinib caused mild effects over cell death (PARP protein cleavage) when used alone in SH-SY5Y cells, but caused the important expressional changes of autophagy proteins LC3, and p62 (Figure 7D, lane 2 versus lane 1). Inhibition of autophagy after combination of entrectinib with LY294002, or 3-MA, was confirmed particularly at the level of p62 protein (Figure 7D, lane 4 and 6 versus lane 2) even though entrectinib maintained its capacity to induce stimulation of LC3-II. In addition, we also revealed the changes in the mTOR-regulating signaling pathway during autophagy activation (Supplementary Figure S4), which suggested that it might be involved in the regulation of this biological process as well. Taken together, these results confirmed a key role of autophagy in helping NB mutated cells to evade apoptosis in the presence of entrectinib, and suggested for the combined treatment of ALK- and autophagic-inhibitors in treatment of NB cells bearing *ALK* gene mutation.

### Autophagy activation is an early event in SH-SY5Y

**Figure 8:**
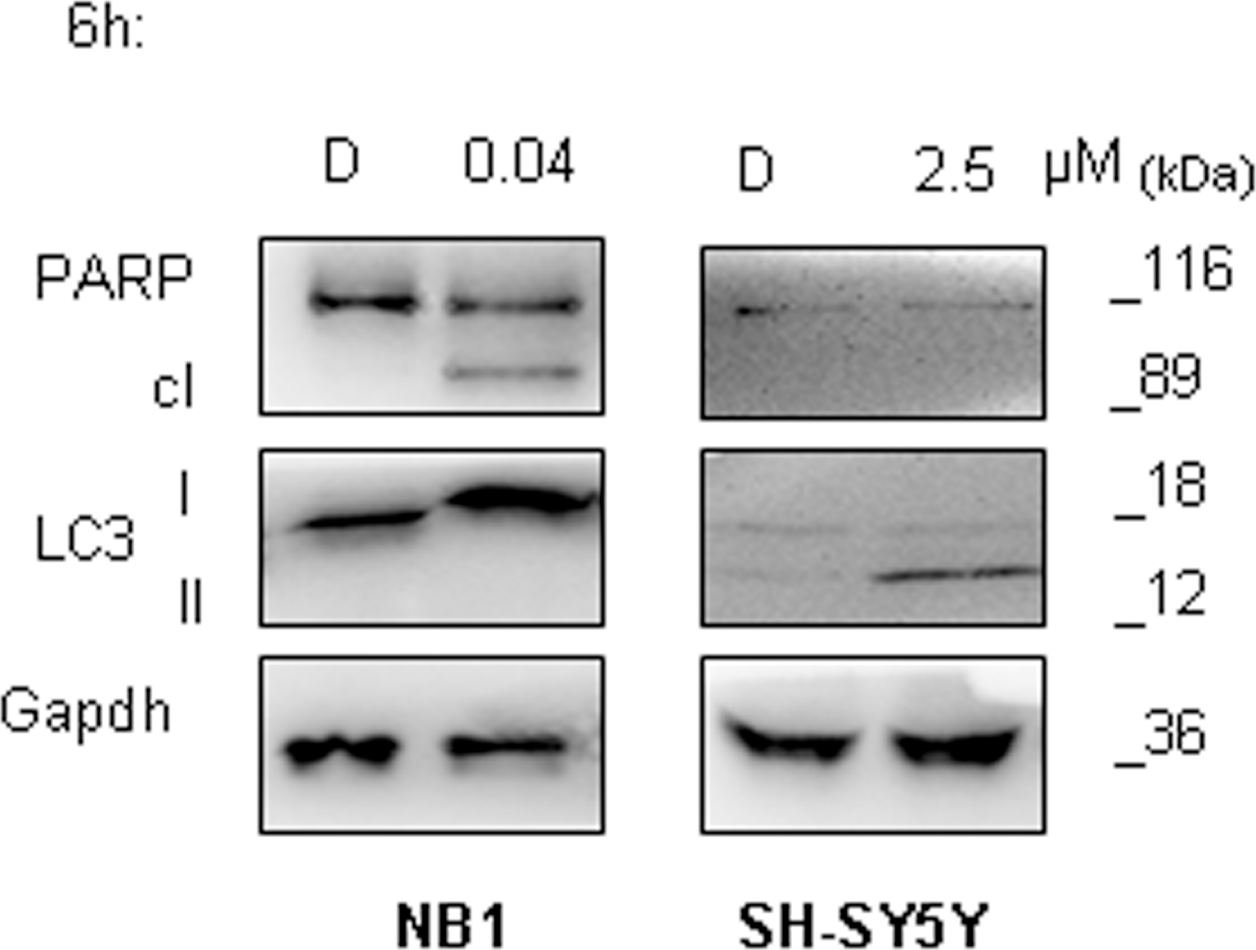
Autophagy activation is an early event in ALK^F1174L^ mutated cells **A.** Western blot analysis was done for the evaluation of LC3 processing, and hence, autophagy activation. NB1 and SH-SY5Y cells were treated shortly (6h) with entrectinib (e), using 0.04 μM, and 2.5 μM, respectively, and in following the expression of LC3-I, and LC3-II proteins was validated. We could confirm autophagy to be an early protective event in SH-SY5Y^F1174L^ cells, being activated after only 6h of treatment with entrectinib. On the contrary, in NB1^amp^ cells, entrectinib showed an immediate apoptosis activation, as confirmed by cleavage of PARP protein, which has not been observed for SH-SY5Y cells. Gapdh was used as protein loading control. D = DMSO.

Additionally, to define timing of autophagy activation after entrectinib administration, we treated SH-SY5Y cells for 6h (5 μM), and analyzed LC3 protein expression afterwards. We could confirm that a protective mechanism in SH-SY5Y cells was activated rapidly in the presence of entrectinib, since LC3-II increased significantly even at this early time point. Autophagy was not observed in NB1 cell line after entrectinib treatment, which was in concordance with other results seen previously for this cell line. On the contrary, NB1 cells were highly sensitive to ALK inhibition attained by entrectinib. We could see that the kinetic of entrectinib in inducing NB1 cell death was quick as well, since 6h treatment provoked a marked PARP cleavage (Figure 8). These findings imply that NB1 are ALK-addicted cells, in which ALK pathway obstruction provokes a destructive cell survival effect.

### Crizotinib causes different autophagy-defined pattern in NB cells with respect to entrectinib, and marginal apoptosis activation

**Figure 9:**
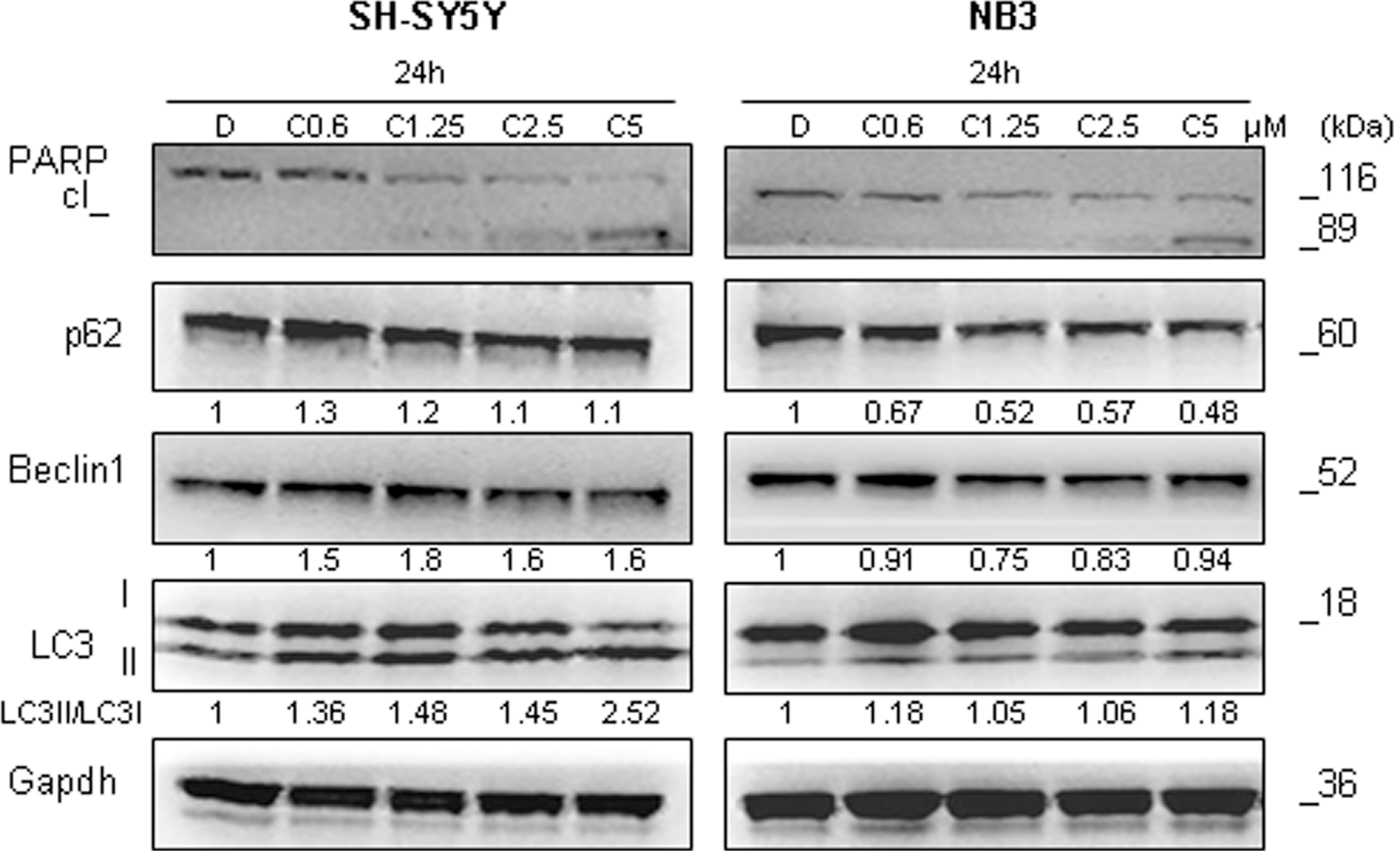
Effects of crizotinib on autophagy induction Immunoblot analyses of autophagy (LC3, p62, and Beclin-1), and apoptosis (PARP) related proteins' expression after treatment with crizotinib are shown. Several concentrations of drug have been applied. Gapdh was used as a control of proper protein loading. Numbers beside letter (c) indicate concentration of crizotinib (μM) used in the experiments. Numbers under the blots indicate relative expression of the protein calculated by densitometry. D = DMSO.

Crizotinib, as a well known anti-ALK drug (PF02341066) was used for the treatment of some tumors, such as a non–small-cell lung cancer with *ALK* rearrangements [25]. However, it was seen that crizotinib can induce autophagy in lung cancer cells, which correlated with resistance to this drug [26]. To define whether crizotinib could provoke autophagy induction in ALK-mutated NB cells, we treated these cells with increasing concentration of crizotinib. After defining IC_50_ (Supplementary Table S6), we selected several concentration under, and above IC_50_ value, treated the cells, and controlled the expression of autophagy related proteins. Among *ALK*-mutated cell lines used for this experiment (NB3 and SH-SY5Y), autophagy induction was more evident for the SH-SY5Y cells (Figure 9). In these cells we observed the increase in LC3-II/LC3-I ratio (LC3-II/LC3-I ratio: DMSO = 1 AU; crizotinib 0.6 μM = 1.36 AU; crizotinib 1.25 μM = 1.48 AU; crizotinib 2.5 μM = 1.45 AU; crizotinib 5 μM = 2.52 AU), slight increase in Beclin-1 level, and almost unchanged expression of p62 protein. In NB3 cell line, there was no significant activation of autophagy after treatment with crizotinib (Figure 9). In addition, similarly to entrectinib, cell lines bearing *ALK* mutations demonstrated PARP cleavage only at the concentrations around defined IC_50_ and above, pointing out that they generally showed poor susceptibility to crizotinib.

## DISCUSSION

Neuroblastoma represents an embryonic tumor which derives from over-proliferating primitive neuroblast cells of the sympathetic nervous system. The most of NB cases are diagnosed during childhood [27]. Around 15 % of all childhood cancer deaths occur due to NB, which makes it a tumor with a great medical, and social impact [28, 29]. Some of the main genes described in NB, *MYCN,* and *ALK*, have been identified as the major pathogenic markers of this malignancy [12]. Therefore, it is understandable why the use of drugs against these markers represents a base for targeted therapy in NB patients. Relatively high frequency of NB patients with deregulated ALK tyrosine kinase rationalizes the ALK-targeting approach, which has been adopted during the recent years [17, 30]. Nonetheless, the ALK-inhibition is found to be a problematic issue due to a rapid resistance development.

Nowadays, the resistance to the kinase inhibitors became one of the most awkward concerns of the oncologists [31]. This is true for the majority of solid tumors, including NBs. In particular, the advanced-stage NB tumors have a high frequency of treatment resistance, urging for innovative, and less aggressive therapies to be defined. To date, several ALK inhibitors have been tested for their efficiency to impede the NB growth, each with different activity [32].

The present study assessed the capability of entrectinib, a novel ALK tyrosine kinase inhibitor, to interfere with cancer cell growth, and proliferation. We tested its influence over viability, and motility of NB cells with various mutation status of *ALK* gene, and validated entrectinib-dependent cell death mediation *in vitro*.

We confirmed that the administration of entrectinib impaired cell proliferation, and abrogated cell-cycle progression, causing the G1-arrest. The rate of cell-cycle blockage after the treatment was concomitant with diminished expression of the *Ki-67,* and with the changes in the expression of the genes involved in cell-cycle regulation. Nevertheless, we delineated a different amplitude of behavior to drug treatment between the ALK amplified or ALK mutated cells, confirming the results discussed by other groups [33]. The results obtained after treatment with entrectinib allowed us to determine that the NB1 cell line was strongly addicted to ALK activity, whereas ALK-mutation bearing cells were less ALK-dependent. In detail, we confirmed the impairment of ALK-downstream signaling molecules, such as pSTAT3, and pERK1/2, in the presence of entrectinib in all cell lines. As a consequence, proliferation of NB cells was impaired, and colony formation capacity was drastically decreased with respect to control treated samples. Also, the motility of the NB cells was significantly inhibited upon addition of entrectinib. However, described cytostatic effects of entrectinib were delineated with diverse intensity among examined NB cell lines.

Furthermore, our data indicated that entrectinib induced Caspase-3 dependent cell death, showing a dose-dependent effect in NB cells. At the protein level, cell mortality induction was confirmed in varying degree between *ALK*-amplified, mutated, or wild-type gene, by studying PARP protein cleavage.

Particular interest in our study was given to understand why the entrectinib efficacy was drastically lower in ALK-mutation bearing cells. We delineated that the mutation occurrence decreased the cell susceptibility to entrectinib through autophagy induction. Treatment of SH-SY5Y^F1174L^ cells with entrectinib resulted in the increased expression of LC3-II, and decreased level of p62 protein, both in time- and dosage-dependent manner. Autophagy was also morphologically characterized by evaluating the autophagosome formation in GFP-LC3 transfected SH-SY5Y cells.

In trying to overcome the observed resistance, and improving the efficiency of entrectinib in these cells, we adopted the combination strategy, using entrectinib together with autophagy inhibitor, Chloroquine (CQ). This treatment rendered the tumor cells prone to death, translating the protective role of autophagy toward apoptosis induction, as confirmed by PARP cleavage, and increased percentage of dead cells in treated samples. These observations allow us to postulate for the existence of alternative signaling pathways which activation might influence a therapy response in NB patients. In various cancers, proteins from the phosphatidylinositol 3-kinase (PI3K)/AKT/mTOR signaling pathway are found altered [33], and have been correlated with autophagy regulation. In our study, we could also confirm the modulation of the mTOR-dependent pathway in SH-SY5Y cells' after entrectinib administration. More precisely, we confirmed that the co-treatment of entrectinib, and Rapamycin, a well-known mTOR inhibitor, was a good strategy to further improve the effects of ALK-inhibitor in NB1 cells. These data support the findings of Moore et al., obtained during examination of crizotinib efficiency in NB [34]. As crizotinib, entrectinib is also a promising ALK inhibitor, and both of drugs are currently in clinical trials for ALK-driven tumors (NCT01154140, and STARTRK-1, respectively). Although both drugs are potent inhibitors of wild-type ALK and ALK fusions, entrectinib is able to inhibit certain ALK mutants that are otherwise resistant to crizotinib [35]. The different sensitivity is likely due to differences in the exact positioning, and bonding of the inhibitor-target interaction between these two compounds (manuscript in preparation). In addition, unlike crizotinib, entrectinib is able to efficiently cross the blood-brain barrier therefore targeting brain tumors and brain metastases [36].

In conclusion, entrectinib inhibited NB cell proliferation, induced cell death, and caused cell cycle arrest, influencing largely the ALK downstream signaling pathway. Our findings identifies entrectinib as a potent inhibitor of ALK-amplified NB1 cells, which seem to be highly dependent on ALK activity, implying for ALK-addiction [37]. On the other side, a low efficiency in ALK-mutated NB cells was supported by the autophagy activation, which was successfully abrogated by combination therapy. In particular, we propose co-treatment of ALK-inhibitor, and CQ, as an effective approach in managing drug resistance, and likely recurrence, or metastasis of NB tumors. The combined treatment strategy may be critical for the increased clinical efficacy of anti-ALK drugs. Our data are in a good agreement with those of Zhang et al and Mitou et al, since they confirm a protective role of autophagy in ALK-dependent tumors other than NB, after use of ALK-inhibitors [26, 38]. Altogether, our data suggest that the pharmacological inhibition of autophagy may be a valuable approach to combat acquired resistance to ALK-tyrosine kinase inhibitor entrectinib, which we propose for a clinical evaluation in NB patients.

## MATERIALS AND METHODS

### Cell culture and treatments

Human NB tumor cell lines NB1, NB3, SH-SY5Y, and IMR32, kindly provided by Dr Luca Longo, from IRCCS AOU San Martino – IST, Genoa, Italy, were cultured in RPMI-1640 medium (Sigma-Aldrich, Milan, Italy) with addition of antibiotics (1%), and fetal bovine serum (FBS; 10%; Gibco, Life Technologies, Monza, Italy). DNA typing for the cell line authentication was done prior the analyses. For each experiment, equal numbers of cells were seeded, and the concentration of DMSO equivalent to those of the chemicals used, was applied to control cells. Entrectinib was provided by Ignyta Inc. (San Diego, CA) under a material transfer agreement; Chloroquine (CQ), 3-Methyladenine (3-MA), Tamoxifen (Tam), Rapamycin (Rapa), Ly294002 (Ly), and bovine serum albumin (BSA) were purchased from Sigma-Aldrich, crizotinib from Selleck Chemicals (Munich, Germany). The concentrations of the compounds used in the study were adapted for each experiment before use.

### Measurement of cell proliferation, viability and mortality

Log-phase cells were seeded into the 96-wells plate (15.000 cells/well), and were allowed for the attachment overnight followed by the treatment with entrectinib (0-10 μM) for 24h, 48h and 72h. Cellular proliferation was quantified with colorimetric methods based on the metabolic reduction of the soluble yellow MTT (3-(4, 5-dimethylthiazol-2-yl)-2, 5-diphenyltetrazolium bromide) dye to its insoluble formazan [39]. Absorbance was red by VICTOR™ Multilabel Plate Reader (PerkinElmer, Waltham, MA) at 486 nm. Results were normalized to time point zero (T= 0h), and calculated for each treatment with respect to DMSO treated controls. Cell viability was determined by trypan blue exclusion assay plating 1 × 10^5^ cells / well in 24 wells plate a day before treatment with entrectinib. The following day, for one randomly selected well, the starting cell number was calculated (T= 0h), whereas to the remaining cells, increasing concentration of entrectinib was administered. Until the 3^rd^ day, every 24h cell number was calculated after trypsinization, centrifugation, and re-suspension in 500 μl of 1xPBS. Staining was done with 0.5% trypan blue. The unstained cells were quantified using the Countess™ automated cell counter (Invitrogen) and final data were calculated with respect to the number of cell determined at time point zero (T= 0h), corresponding to the value 1 on the graph. Cell death was measured by flow cytometer Cytomics FC500 (Beckman Coulter, Brea, CA) using the Apodirect Kit (BD Bioscience, San Diego, CA). Fluorescein labeled TdT-mediated dUTP nick end labeling (TUNEL) assay allowed distinction of dying NB cells after treatment with entrectinib. Apoptosis was measured by flow cytometry measuring activation of Caspase-3, one of the hallmarks of early apoptotic events. Preparation of the cells for analysis was done as suggested by manufacturer.

### RNA isolation, quantitative real-time polymerase chain reaction, and reverse-transcription polymerase chain reaction

Total cellular RNA was extracted by TRIzol reagent (Invitrogen, Life Technologies), as indicated by the manufacturer and the quality was controlled on Agilent 2100 Bioanalyzer (Agilent Technologies, Tokyo, Japan). One mg of total RNA was reversely transcribed using random hexamers, and the Superscript II (Invitrogen) according to the manufacturer's instructions. Gene expression was evaluated by quantitative PCR (qRT-PCR; Applied Biosystems 7900HT Fast Real Time PCR System) using SYBR Green PCR Master Mixture Reagents (Applied Biosystems, Forest City, CA). The primer sets are available upon request. Gapdh was used to normalize levels of mRNA for the relative quantification method of analysis. qRT-PCR reactions (20 μL) were carried out as triplicate reactions, and final data were calculated from three separate experiments. Relative quantification analysis was done using the comparative ddCt method [40]. Experimental data were expressed as the mean ± SEM of the n-fold change from at least three independent experiments.

### Wound healing assay

The total of 3×10^4^ cells (80% confluence) were plated within each of the two cell culture reservoirs separated by a 500 μm thick silicone wall (IBIDI, Milano, Italy). Day after, the silicone insert was removed from the surface and treatments with entrectinib or DMSO vehicle were performed on four different human NB cell lines. The cells were allowed to grow for another 48-72h, and images were taken every 24h. Images were taken by Nikon Eclipse TS100 microscope (NiKon Eclipse TS 100, Southern Micro Instruments, Marietta, GA) with a Nikon Coolpix camera attached to the microscope. Wound healing was analyzed by MyWim software provided by IBIDI [41] for each time point. Data were presented as percentage of scratch area obtained for 3 separate measurements as mean ± SEM.

### Western blot analysis

Samples containing 100 μg of total proteins (for ALK detection), or 50 μg (for proteins other than ALK) were lysed with commercially available lysis buffer (Biosource International; Camarillo, CA), and analyzed with SDS-polyacrylamide gel electrophoresis (SDS-PAGE) using precast gradient polyacrylamide gels (Bio-Rad, Milano, Italy). Separated proteins were transferred onto PVDF membranes, by using a semidry transfer cell (BioRad, Trans-blot SD), and blocked with 5 % BSA in 1xPBS for 1h at room temperature. The membranes were probed with monoclonal antibody, at a dilution suggested by the manufacturer, overnight at 4°C. Following primary antibodies were used: anti-PARP, anti-ERK1/2, anti-pERK1/2, anti-STAT3, anti-pSTAT3, and anti-pALK (Cell Signaling, Danvers, MA), anti-LC3, anti-Beclin-1, and anti-Gapdh, (Novus Biologicals, Littleton, CO), anti-p62/SQSTM1 (Abnova, Taipei City, Taiwan), anti-ALK (Abcam, Cambridge, UK).

In following, the membranes were incubated with a horseradish peroxidase-conjugated secondary antibody (Santa Cruz Biotechnology, Santa Cruz, CA) for 1h at room temperature, and the immunoreactive bands were detected with chemiluminescence substrate kit (ECL advance; Amersham Pharmacia Biotec, Piscataway, NJ) under the Alliance imaging system (UVItec, Cambridge, UK). Re-probing of a blot with new primary antibodies was done after the stripping of antigen-antibody complex using a mild stripping solution (0.2 M glycine-HCl, pH 2.2, 0.1% SDS, 0.01% Tween-20) for 1h at room temperature, followed by 3 washing with 1xPBS with 0.1% Tween-20 (Sigma-Aldrich). The membranes were then blocked as described, and re-probed with primary, and secondary antibodies as described.

### Colony formation assay

Two thousand cells of each NB cell type were seeded in a 24 wells plate using MethoCult semi-solid medium (Stemcell Technologies, Milan, Italy), with the addition of entrectinib (concentration adapted for each cell type) or control DMSO vehicle, and cells were left to grow for 2 weeks. Colonies (foci) were visualized after being stained with MTT for 4h. Pictures of the colonies were taken under light microscope, and total colony number was determined for each well by ImageJ software [42]. Three independent experiments were done for each cell type.

### Cell cycle analysis

Analysis of cell cycle was done by flow cytometry. NB cells were seeded in 6 wells plate at an initial density of 8×10^5^ cells per well. Cells were left to adhere before being treated with entrectinib. At the end of treatment (24h), the cells were harvested by trypsinization, washed, fixed after centrifugation in 2ml of 70% Ethanol and left at −20°C until analysis. After DNA staining by Propidium iodide (Sigma-Aldrich) in addition of RNase (Qiagen, Milan, Italy) for 30 min at room temperature in the dark, the cell-cycles of the samples were measured on a FACScalibur cytometer (Becton-Dickinson, Franklin Lakes, NJ). Analysis was performed with the CellQuest software, and presented as percentage of the cells in each phase of cell-cycle (G1, S and G2/M).

### Immunofluorescence imaging

Cells were fixed in cold 4% formaldehyde washed in 1x phosphate-buffered saline (PBS), and permeabilized in 0.1 % Triton-X 100 (Sigma-Aldrich, diluted in PBS) for 10 min. After blocking with 5 % BSA in PBS for 30 min, samples were washed thrice in 1xPBS and incubated with rabbit-polyclonal anti-Ki-67 primary antibody (DakoCytomation, Carpinteria, CA) at 4°C overnight. After washing in 1xPBS, the incubation with Alexa Fluor 594-conjugated goat anti-rabbit immunoglobulin G (red; 1:2.000, Life technologies, Monza, Italy) was done. Cell nuclei were counterstained with 40, 6-diamidino-2-phenylindole (DAPI; blue; 1:10.000; Sigma-Aldrich) diluted in 1xPBS for 15 min. Stained cells were mounted in Vector Shield mounting medium (Vector Laboratories LTD, Peterborough, UK), and images were examined under a fluorescence microscope at 60X (Vico, Eclipse Ti80, Nikon, Tokyo) or 100X (Carl Zeiss Microscopy, Germany) objective lens magnification equipped with a digital camera. Laser power, optimized on control-stained cells, remained the same throughout each experiment.

### Transient transfections with GFP-LC3 plasmid

Plasmid carrying a construct for a green fluorescent protein labeled to autophagosome-associated LC3 protein (GFP-LC3) was kindly provided by Dr Leonardo Salviati research group from the IRP-Città della Speranza, Padua, Italy. The SH-SY5Y cells were transiently transfected using Effectene Transfection Reagent (Qiagen) following manufacturer procedure, with 2–4 μg of plasmid DNA in 24 wells plate. After 24h, entrectinib was added (5 μM), and 24h later immunocytochemistry was performed using DAPI to mark cells' nuclei. The localization of GFP-LC3 within SH-SY5Y transfected cells was determined by fluorescence microscopy.

### Statistical analyses

All experiments were repeated independently at least three times, and the experimental data were expressed as the mean ± SEM. Statistical analyses were conducted using Student's t-test. Group differences resulting in p values of ≤ 0.05 were considered to be statistically significant, and marked with the asterisk *p on the graphs. All analyses were carried out with the GraphPad Prism program (GraphPad Software, Inc., San Diego, CA). Error bars on all graphs consider 95% confidence intervals.

## SUPPLEMENTARY FIGURES AND TABLES


