# Supplementary material for: Combating autophagy is a strategy to increase cytotoxic effects of novel ALK inhibitor entrectinib in neuroblastoma cells
Source: Oncotarget. 2015 Dec 28;7(5):5646–63. doi: 10.18632/oncotarget.6778 (PMC4868711; doi:10.18632/oncotarget.6778)
Supplement: Supplementary file 1 [file oncotarget-07-5646-s001.pdf]

SUPPLEMENTARY FIGURES AND TABLES

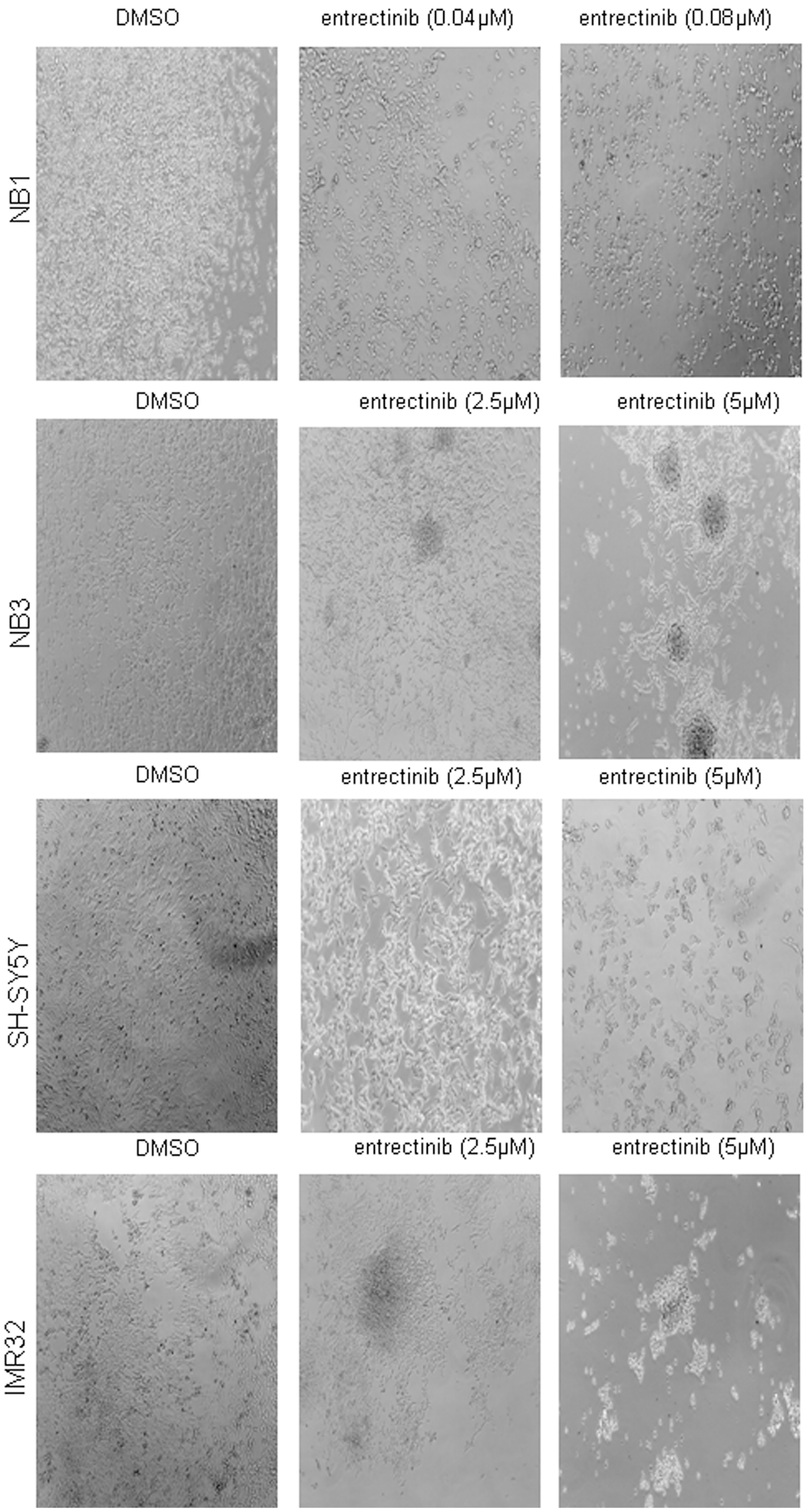

Supplementary Figure S1: Morphological alterations in NB cell lines treated with entrectinib. NB cell lines, NB1, NB3, SH-SY5Y and IMR32, were treated with 2 different concentrations of entrectinib (0.04 and 0.08 μM, or 2.5 and 5 μM). Cell morphology after treatment with increasing concentration of entrectinib has been controlled 72 h post-treatment. Light microscopy images were taken at 10X magnification. Evident decrease in cell number, and severe changes in cell morphology can be noted from the presented images. DMSO vehicle treated cells were used as controls.

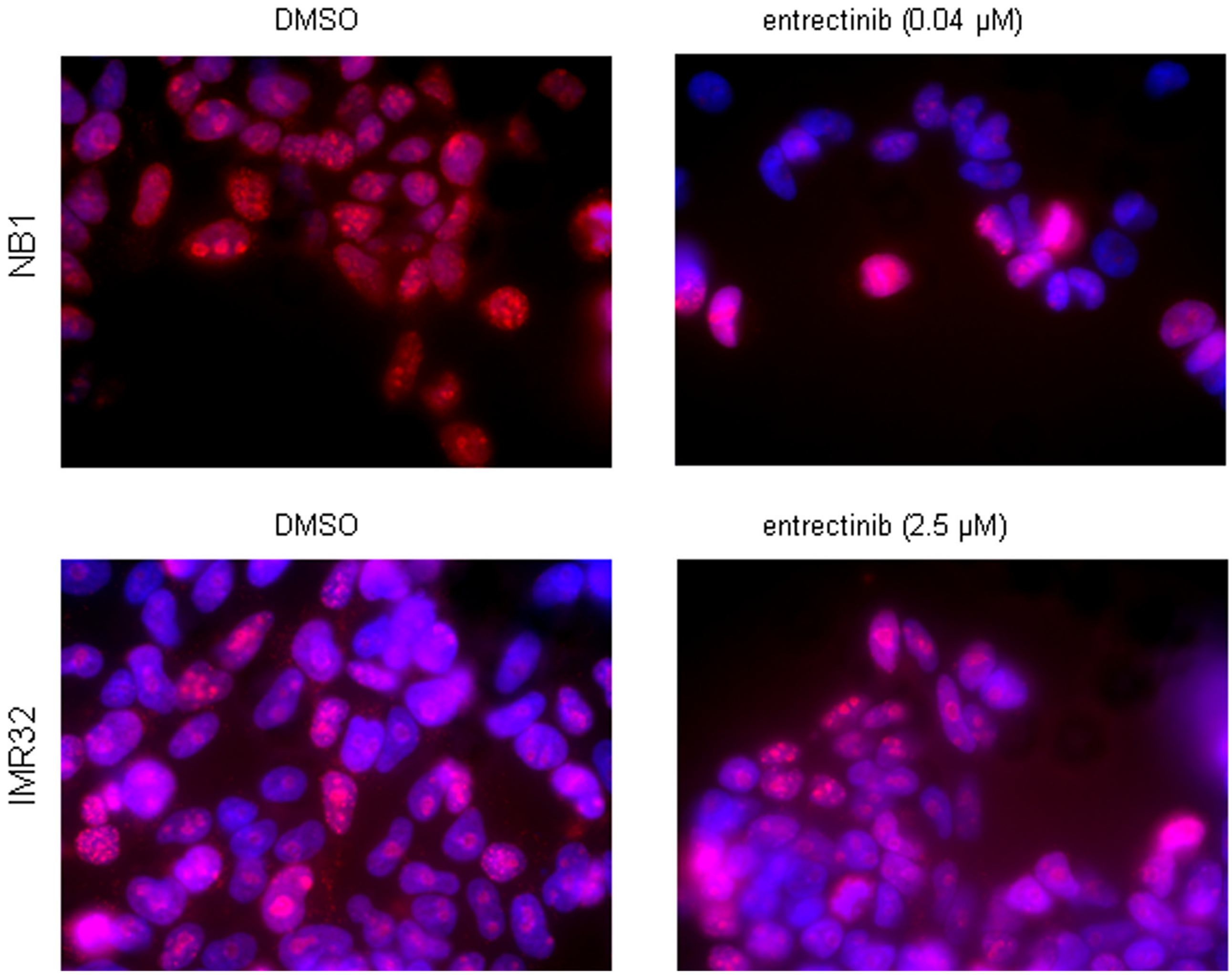

Supplementary Figure S2: Immunofluorescence analysis of Ki-67 protein expression. NB1, and IMR32 cells were used to validate the expressional changes observed previously for *Ki-67* mRNA level. The changes in Ki-67 protein expression (red signal) were congruent with the decrease in mRNA level measured by qRT-PCR analysis. Cell nuclei were counterstained with DAPI (blue). Images were taken on a confocal microscope (60X immersion objective), equipped with a digital camera. Numbers within the brackets indicate the μM of entrectinib used in the experiment.

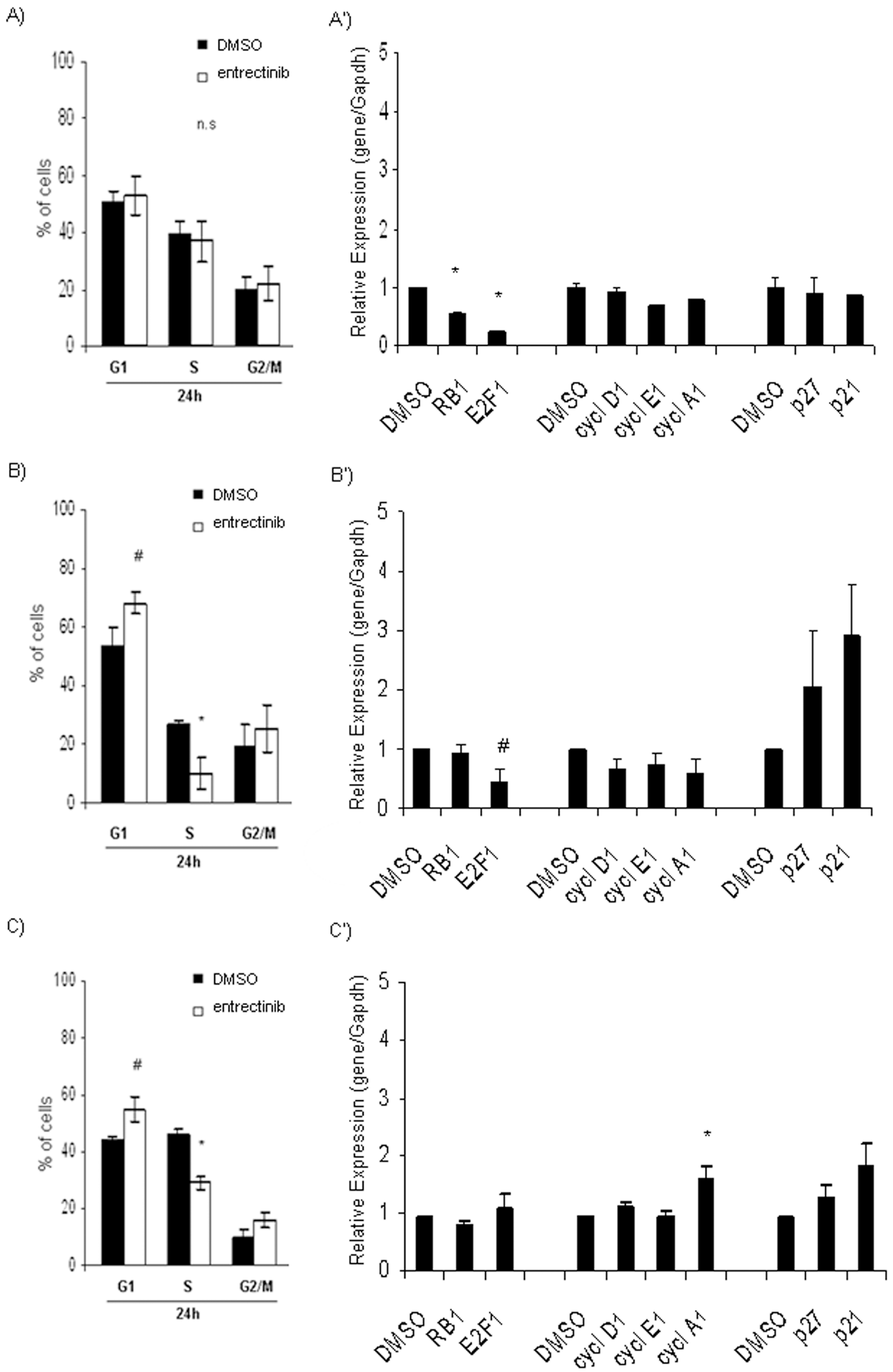

Supplementary Figure S3: Cell-cycle changes after use of entrectinib. A-C. Cell cycle profile was evaluated by flow cytometry after use of IC_{50} of entrectinib. Percentage of cells in each phase of cell cycle is presented. Similar to NB1 cells (Figure 2A), a tendency for the G1-phase accumulation was seen in NB3, SH-SY5Y, and IMR32 cell lines. Results were considered significant for *$p \leq 0.05$. n.s: not-significant; #: close to significance. A'-C') qRT-PCR was performed for the evaluation of mRNA levels of the main cell-cycle regulators *RB1, E2F1, Cyclin A1, D1, E1, p21, and p27*, after treatment with entrectinib (2.5 µM for NB3, SH-SY5Y, and IMR32). Data were normalized for *Gapdh* internal control, and calculated with respect to the relative expression (RQ) determined for DMSO control samples (RQ^{DMSO} = 1). Results were considered significant for $p \leq 0.05$, and indicated with an asterisk (*).

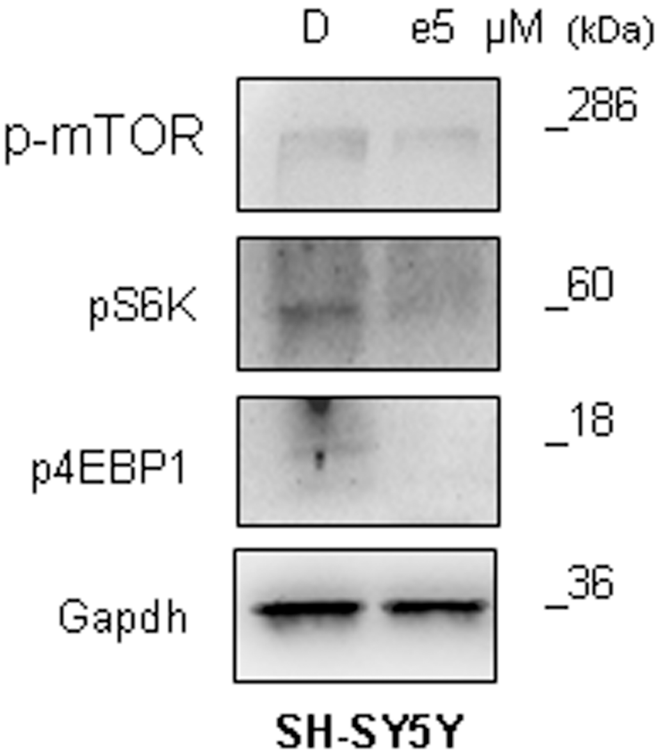

Supplementary Figure S4: mTOR pathway during autophagy activation. A. Immunoblot analysis was done after treatment with entrectinib (e; 5 μM) to evaluate the eventual expressional changes in mTOR-related protein signaling. Observed decrease suggested for the importance of mTOR signaling at the autophagy regulation in SH-SY5Y cells. Gapdh was used as a protein loading control. D = DMSO.

Supplementary Table S1: Entrectinib Inhibitory concentration 50% (IC_{50}) for NB cell lines

The concentration of entrectinib that led to 50% decrease in cell proliferation was determined by MTT assay, and presented for 3 time points, 24 h, 48 h, and 72 h. The results represents mean ± SEM for minimum of three independent experiments obtained for the same number of cells. Four cell lines were used, each with different status of ***ALK*** gene: $NB1^{amp}$; $NB3^{R1275Q}$; $SH\text{-}SY5Y^{F1174L}$, and $IMR32^{wt}$.

Cell lines^{ALK}	IC_{50} mean ± SEM (μM)
**$NB1^{amp}$**	
24 h	0.31 ± 0.05
48 h	0.035 ± 0.009
72 h	0.03 ± 0.01
**$NB3^{R1275Q}$**	
24 h	2.65 ± 1.39
48 h	2.24 ± 0.89
72 h	3.27 ± 0.27
**$SH\text{-}SY5Y^{F1174L}$**	
24 h	4.34 ± 1.18
48 h	3.32 ± 0.90
72 h	2.42 ± 1.34
**$IMR32^{wt}$**	
24 h	3.68 ± 0.91
48 h	3.29 ± 0.35
72 h	3.06 ± 0.61

Supplementary Table S2: Trypan blue exclusion assay

A fold change in cell number was evaluated after treatment with increasing concentration of entrectinib. The cells were counted by trypan blue, and data normalized to the cell number measured at the moment of drug addition (***T*** = 0 h). Results were obtained for at least three independent measurements. * - indicates statistically significant difference in cell number fold change with respect to DMSO control, with the following ***p*** values at 72 h time point: NB1 $p < 0.005$; NB3 $p < 0.05$; SH-SY5Y $p < 0.05$; IMR32 $p < 0.05$.

Cell lines^{ALK}	Treatment and concentration of entrectinib	24 h Cell number fold change(mean ± SEM)	48 h Cell number fold change(mean ± SEM)	72 h Cell number fold change(mean ± SEM)
**NB1^{amp}**	DMSO	3.64 ± 0.39	5.24 ± 0.87	8.65 ± 0.65
	0.04 μM	2.79 ± 0.37	2.86 ± 0.37	2.76 ± 0.62*
	0.08 μM	2.42 ± 0.25	2.82 ± 0.12	2.75 ± 0.11*
	0.16 μM	2.46 ± 0.60	2.36 ± 0.40	2.30 ± 0.56*
**NB3^{R1275Q}**	DMSO	3.61 ± 0.77	7.92 ± 1.51	8.36 ± 1.42
	2.5 μM	2.74 ± 0.67	2.34 ± 0.13	2.86 ± 0.93*
	5 μM	2.11 ± 0.31	2.94 ± 1.17	2.44 ± 0.46*
	7.5 μM	1.51 ± 0.51	2.22 ± 1.00	2.02 ± 0.02*
**SH-SY5Y^{F1174L}**	DMSO	2.59 ± 0.49	4.28 ± 0.58	4.89 ± 1.36
	2.5 μM	2.33 ± 0.46	2.43 ± 0.17	3.20 ± 0.26
	5 μM	1.32 ± 0.40	2.02 ± 0.16	2.04 ± 0.24
	7.5 μM	0.81 ± 0.58	0.57 ± 0.40	0.70 ± 0.42*
**IMR32^{wt}**	DMSO	2.95 ± 0.83	5.10 ± 1.57	8.30 ± 1.51
	2.5 μM	3.29 ± 0.90	2.13 ± 0.65	5.26 ± 1.42
	5 μM	3.65 ± 0.95	2.12 ± 0.69	3.07 ± 1.05*
	7.5 μM	2.79 ± 0.70	2.09 ± 0.79	1.67 ± 1.21*

Supplementary Table S3: Relative gene expression (RQ) after 24h of treatment with entrectinib in NB1 cell line

qRT-PCR data obtained for the ***RB1, E2F1, Cyclin A1, D1, E1, p21, and*** p27 (Figure 2B, and Supplementary Figure 3)**,** were presented as a mean ± SEM for at least 3 independent treatments. Data were normalized for ***Gapdh*** internal control, and calculated with respect to the relative expression (RQ) determined for DMSO control samples ($RQ^{DMSO} = 1$). ***p*** values are indicated within the table. n.s: not-significant

Gene	Relative Expression (RQ)mean ± SEM	p value
RB1	0.47 ± 0.14	0.02
E2F1	0.17 ± 0.06	0.0005
CyclD1	0.67 ± 0.13	0.06
CyclE1	0.48 ± 0.05	0.0001
CyclA1	0.16 ± 0.03	0.0001
p27	2.01 ± 0.70	n.s.
p21	1.46 ± 0.12	0.02

Supplementary Table S4: Caspase-3 activation after addition of entrectinib
Results shown here represent the percentage of Caspase-3 positive cells, measured after treatment with the indicated entrectinib concentrations (µM), by flow cytometry approach. The results represent mean ± SEM of three independent experiments. DMSO treated cells were used as controls. ***p*** value are indicated within the table. n.s: not-significant.

Cell lines^{ALK}	Treatment and concentration of entrectinib	24 h (mean ± SEM)%	*p* value
**NB1^{amp}**	DMSO	6.2 ± 1.3	
	0.04 µM	11.5 ± 1.4	0.03
	0.08 µM	14.2 ± 2.8	0.04
	0.1 µM	14.6 ± 2.5	0.04
**NB3^{R1275Q}**	DMSO	4.7 ± 1.6	
	2.5 µM	7.9 ± 3.2	n.s.
	5 µM	9.7± 2.5	n.s.
	7.5 µM	16.2 ± 5.5	n.s.
**SH-SY5Y^{F1174L}**	DMSO	5.7 ± 1.0	
	2.5 µM	4.9 ± 0.9	n.s.
	5 µM	10.1 ± 1.3	0.04
	7.5 µM	15.1 ± 0.6	0.002
**IMR32^{wt}**	DMSO	9.5 ± 1.5	
	2.5 µM	15.5 ± 1.2	0.01
	5 µM	15.8 ± 2.1	0.03
	7.5 µM	23.2 ± 1.8	0.001

Supplementary Table S5: Cell death activation after addition of entrectinib

Results present percentage of dead cells, obtained by TUNEL assay, applying flow cytometry technique. The results refer to 3 different concentrations (μM) of entrectinib listed in the table. DMSO treated cells were used as control samples. ***p*** values are indicated within the table. n.s: not-significant.

Cell lines^{ALK}	Treatment and concentration of entrectinib	24 h (mean ± SEM)%	*p* value
**NB1^{amp}**	DMSO	3.5 ± 1.1	
	0.04 μM	8.2 ± 3.8	0.04
	0.08 μM	10.3 ± 3.2	0.03
	0.16 μM	29.6 ± 4.5	0.0001
**NB3^{R1275Q}**	DMSO	4.1 ± 2.1	
	2.5 μM	15.3 ± 1.8	0.02
	5 μM	23.4 ± 0.7	0.001
	7.5 μM	45 ± 3	0.0004
**SH-SY5Y^{F1174L}**	DMSO	4.5 ± 0.8	
	2.5 μM	11.3 ± 3.6	n.s.
	5 μM	22.4 ± 0.1	0.0001
	7.5 μM	46.3 ± 8.7	0.009
**IMR32^{wt}**	DMSO	8.2 ± 1.2	
	2.5 μM	14.3 ± 4.1	n.s.
	5 μM	21.9 ± 2.4	0.007
	7.5 μM	21.2 ± 2.0	0.005

Supplementary Table S6: Time dependent Inhibitory concentration 50 % (IC50) for crizotinib in neuroblastoma cell lines
The concentration of crizotinib that led to 50% decrease in cell proliferation was determined by MTT assay, and presented for 3 time points, 24 h, 48 h, and 72 h. The results represents mean ± SEM for minimum of three independent experiments obtained for the same number of cells. Four cell lines were used, each with different status of ***ALK*** gene: NB1^{amp}; NB3^{R1275Q}; SH-SY5Y^{F1174L}, and IMR32^{wt}.

**Cell lines^{ALK}**	**IC_{50}mean ± SEM (µM)**
**NB1^{amp}**	
24 h	0.59 ± 0.23
48 h	0.36 ± 0.43
72 h	<0.03125
**NB3^{R1275Q}**	
24 h	2.21 ± 0.28
48 h	0.77 ± 0.20
72 h	<0.5
**SH-SY5Y^{F1174L}**	
24 h	1.6 ± 0.7
48 h	1.34 ± 0.60
72 h	1.1 ± 0.7
**IMR32^{wt}**	
24 h	2.19 ± 1.19
48 h	0.71 ± 0.03
72 h	0.64 ± 0.06
